# A Direct Analysis of β-*N*-methylamino-l-alanine Enantiomers and Isomers and Its Application to Cyanobacteria and Marine Mollusks

**DOI:** 10.3390/toxins15110639

**Published:** 2023-11-01

**Authors:** James S. Metcalf, Sandra Anne Banack, Peter B. Wyatt, Peter B. Nunn, Paul A. Cox

**Affiliations:** 1 Brain Chemistry Labs, Box 3464, Jackson, WY 83001, USA; sandra@ethnomedicine.org (S.A.B.); paul@ethnomedicine.org (P.A.C.); 2 Department of Biological Sciences, Bowling Green State University, Bowling Green, OH 43403, USA; 3 The School of Physical and Chemical Sciences, Queen Mary University of London, London E1 4NS, UK; p.b.wyatt@qmul.ac.uk (P.B.W.); p.nunn@qmul.ac.uk (P.B.N.)

**Keywords:** BMAA, enantiomers, cyanobacteria, neurological disease, chirality

## Abstract

Of the wide variety of toxic compounds produced by cyanobacteria, the neurotoxic amino acid β-*N*-methylamino-l-alanine (BMAA) has attracted attention as a result of its association with chronic human neurodegenerative diseases such as ALS and Alzheimer’s. Consequently, specific detection methods are required to assess the presence of BMAA and its isomers in environmental and clinical materials, including cyanobacteria and mollusks. Although the separation of isomers such as β-amino-*N*-methylalanine (BAMA), *N*-(2-aminoethyl)glycine (AEG) and 2,4-diaminobutyric acid (DAB) from BMAA has been demonstrated during routine analysis, a further compounding factor is the potential presence of enantiomers for some of these isomers. Current analytical methods for BMAA mostly do not discriminate between enantiomers, and the chiral configuration of BMAA in cyanobacteria is still largely unexplored. To understand the potential for the occurrence of D-BMAA in cyanobacteria, a chiral UPLC-MS/MS method was developed to separate BMAA enantiomers and isomers and to determine the enantiomeric configuration of endogenous free BMAA in a marine *Lyngbya* mat and two mussel reference materials. After extraction, purification and derivatization with *N*-(4-nitrophenoxycarbonyl)-l-phenylalanine 2-methoxyethyl ester ((*S*)-NIFE), both L- and D-BMAA were identified as free amino acids in cyanobacterial materials, whereas only L-BMAA was identified in mussel tissues. The finding of D-BMAA in biological environmental materials raises questions concerning the source and role of BMAA enantiomers in neurological disease.

## 1. Introduction

Cyanobacteria are well known for producing a wide array of toxic compounds, both with acute and chronic toxicities [1,2]. With fossils dating back up to 3.6 billion years [3,4,5,6,7], these organisms are considered to have had a large influence on the evolution of our planet, such as through the generation of the oxygen atmosphere and the Great Oxidation Event [8,9,10,11]. More recently, cyanobacteria have also been shown to produce a large number of toxic compounds [12,13,14,15], including peptides, alkaloids and components of the cell wall, such as microcystins [16,17,18,19,20], cylindrospermopsins [21,22,23,24,25,26], anatoxin-a [27,28,29,30], anatoxin-a(S) (guanitoxin; [31,32,33,34,35,36,37]), saxitoxins [38,39,40,41,42], and LPS [43,44,45] as examples. With regard to human health, exposure to these toxins can occur through a wide range of exposure routes such as drinking water [46,47], the consumption of seafood including fish and shellfish [48,49], administered medicinal water, often via intravenous injection [50,51], and through air during the process of inhalation [52,53,54] as examples.

As the potential for toxin exposures and the associated risks are increasingly being better understood, accurate assessments using a variety of methods and procedures are increasingly required to protect human and animal health. In order to achieve this, the use and application of analytical methods for the detection of toxins is essential for risk assessments concerning the potential for adverse health effects. This has resulted in the capacity to detect a wide variety of possible cyanobacterial toxins that are present, with a diverse and adaptable set of analytical methods that continue to be developed and refined. For the assessment of acutely toxic cyanobacterial products, such as microcystins, anatoxin-a, guanitoxin and saxitoxins, a wide range of methods have been employed. These include methods for the potential for toxin production, such as PCR [55,56,57] and microscopy [58,59], to detect the producer organisms or genes concerning the production of the toxin molecular machinery. Conversely, depending on the scenario, the assessment of actual toxins may be required. A wide variety of biochemical-based analytical techniques have been developed to permit this, ranging from bioassays [60,61,62,63], protein binding and enzyme inhibition assays [64,65,66,67,68,69], through to immunoassays [70,71,72,73,74,75,76]. Increasingly, physicochemical methods are being employed to measure these toxins in a wide range of matrices. Although UV methods have been successfully employed for their detection, they are increasingly being replaced with mass spectrometry. Using single or triple quadrupole mass spectrometry, the ability to detect, e.g., microcystins [77,78], cylindrospermopsin [79] and anatoxin-a [80], is possible. In cases where acute toxicity occurs, a retrospective assessment of toxin types, exposure routes and concentrations is generally easy to carry out if investigators are aware of the potential for cyanobacterial toxins as the proximal cause of the intoxication. Increasingly, however, the possibility exists that long-term chronic exposures to cyanotoxins may be a cause for concern. Certainly, the association between microcystin and nodularin as potential tumor promoters and carcinogens [81] shows that long-term human health could be adversely affected, and increasingly, chronic exposure to saxitoxin may be connected to cognitive decline [82].

**Figure 1:**
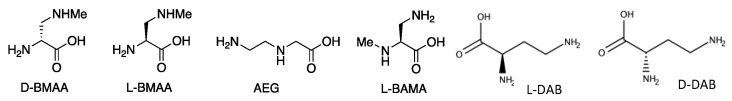
Structures of L- and D-BMAA and related isomers.

In addition to the various cyanobacterial peptides and alkaloids with associated neuro-, hepato- and cytotoxicities, research into neurotoxic amino acids and their relationship to human neurodegenerative disease has continued apace (e.g., [83]), largely investigating β-*N*-methylamino-l-alanine (BMAA) and its isomers (Figure 1), the achiral *N*-(2-aminoethyl)glycine (AEG), l-2,4-diaminobutyric acid (DAB) and β-amino-*N*-methyl-l-alanine (BAMA) [84]. The need to analyze a wide variety of sample types has led to the introduction of a number of analytical methods to specifically identify BMAA and its isomers in a range of clinical and environmental matrices [85]. Analytical methods and laboratory equipment have progressed from fluorescence to mass spectrometric detection, with or without chemical derivatization to provide accurate and specific detection of these molecules [85]. Although such methods are useful for isolating and quantifying BMAA and its isomers, so far, methods to understand the enantiomers of such compounds are limited [86,87,88].

BMAA was originally isolated from an ethanolic extract of cycad seeds, and an optical analysis of the compound determined the presence of the L-enantiomer [89,90]. Toxicity studies with the L-enantiomer of BMAA showed toxicity when compared with DL-BMAA administered to chicks at acute doses [91]. During digestion, changes in pH and temperature can affect the configurations of chiral molecules such as amino acids [92]. Furthermore, in mammals, D-amino acids such as D-serine and D-aspartic acid do occur as free compounds, often involved in neurotransmission [93,94,95]. Being true bacteria, cyanobacteria are capable of producing a wide range of D-amino acids, often present in peptides [1], and along with the analysis of plants, over 800 novel amino acids are currently known to exist [96].

A variety of methods have been introduced for the measurement of BMAA in a wide range of samples and matrices. These methods are generally split based upon whether BMAA is measured directly or after chemical modification. Direct analyses usually employ hydrophilic interaction liquid chromatography (HILIC), whereas chemical modifications can include 9-fluorenylmethoxy carbonyl (fmoc), propyl chloroformate (PCF), dansyl chloride (DC) and 6-aminoquinolyl-*N*-hydroxysuccinimidyl carbamate (AQC) to increase the size and detectability of the molecule using reverse-phase liquid chromatography [85]. The current advantages of the HILIC methodology allow for direct analysis without any chemical modification, but do require specialized chromatographic columns. However, the ZIC-HILIC column has been demonstrated to be unreliable for BMAA analysis, and further systematic method validations are needed [97]. The chemical derivatization of BMAA, although adding an additional preparation step, does allow for the use of common C18 stationary phases, and in general, results in greater sensitivity and detectability, with robust validation solidly demonstrated [85,98,99]. BMAA has been analytically detected in cyanobacteria [87,100] and is considered to also be produced by diatoms and possibly dinoflagellates [101,102,103,104,105,106], although little research has been performed on the enantiomers that are present in these organisms. Previous methods have separated synthetic L- and D-BMAA standards using derivatized [86,88] and underivatized [87] methods. The presence of BMAA enantiomers has been investigated in cycads [87,88], cyanobacteria and in mammalian liver, blood and CNS after dosing with synthetic L-BMAA [87]. Although the presence of D-BMAA has not so far been widely reported, this enantiomer has been shown to be neurotoxic and was found in the central nervous system of mammals after dosing with L-BMAA [87,107]. Consequently, if D-BMAA was present in cyanobacterial bloom samples and people or animals were exposed, then synergistic toxicological effects may potentially result from the presence of both enantiomers, in addition to the presence of toxic isomers [107].

A number of techniques are available for the separation of amino acid enantiomers, using underivatized methods such as crown ether columns and other specialized UPLC or HPLC stationary phases (e.g., [108]). Alternatively, a wide variety of derivatizing reagents are available, such as Marfey’s reagent and chloroformates, that can be used to chemically modify the compound, generally by inserting a second chiral center into the molecule, allowing for the easier separation of stereoisomers (e.g., [88]). Further chiral derivatization reagents include (*S*)-NIFE (*N*-(4-nitrophenoxycarbonyl)-l-phenylalanine 2-methoxyethyl ester), which has been successfully applied to the detection of amino acid enantiomers in a range of biological materials such as urine [109].

Using underivatized chiral techniques, L-BMAA has been identified in *Nostoc* sp. isolated from Guamanian cycads [87]. Although this method allowed for the fractionation and semi-purification of the extracts, it is more labor- and time-intensive than direct derivatization and analysis. Therefore, the purpose of this study was to examine the free amino acid fraction of a marine *Lyngbya* mat and mussel tissues known to contain BMAA to determine the enantiomeric configuration of free BMAA.

## 2. Results

**Figure 2:**
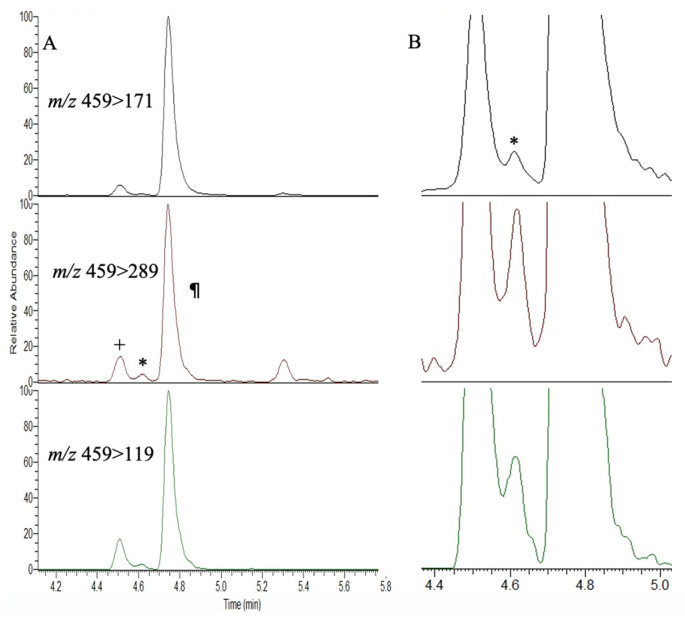
AQC derivatization and LC-MS/MS analysis of an extract of *Lyngbya* spp. for the presence of BMAA (*) and isomers AEG (+) and DAB (¶) with their daughter ions at *m*/*z* 459 > 171, 459 > 289 and 459 > 119. (**A**) Full chromatogram; (**B**) expanded view showing the BMAA peak at 4.64 min (*).

The acid hydrolysis of the *Lyngbya* mat material showed the presence of AEG, BMAA, and DAB (Figure 2), with a total (free + bound) BMAA concentration of 460 ng/g when derivatized with AQC.

**Figure 3:**
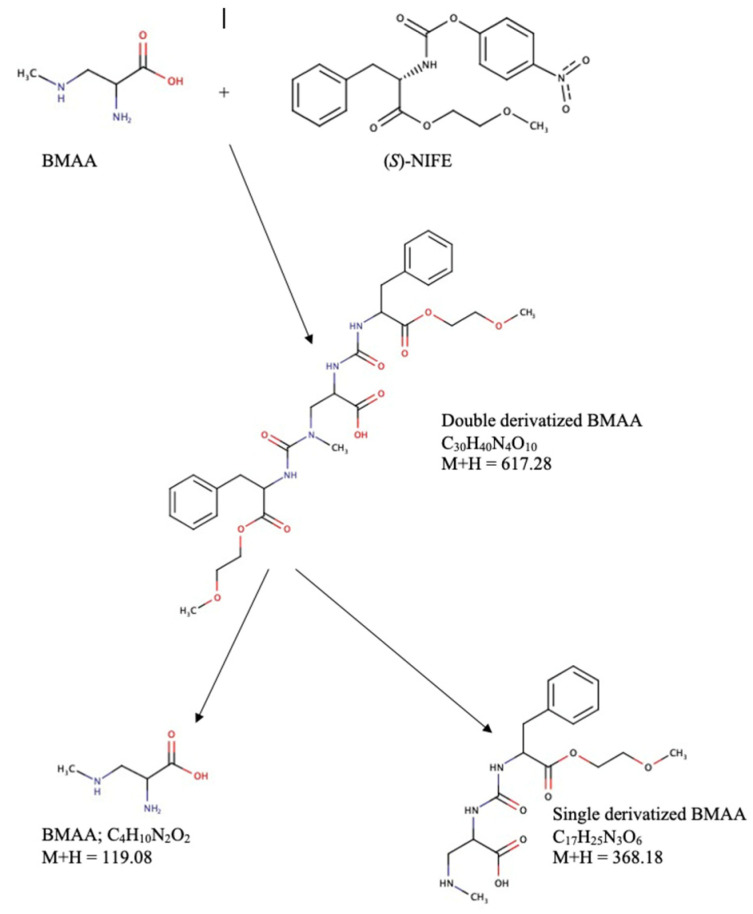
Schematic representation of (*S*)-NIFE derivatization of BMAA showing breakdown products observed in LC-MS and LC-MS/MS.

For the assessment of free enantiomers, a new method was developed to derivatize the enantiomers and isomers with (*S*)-NIFE (Figure 3).

**Figure 4:**
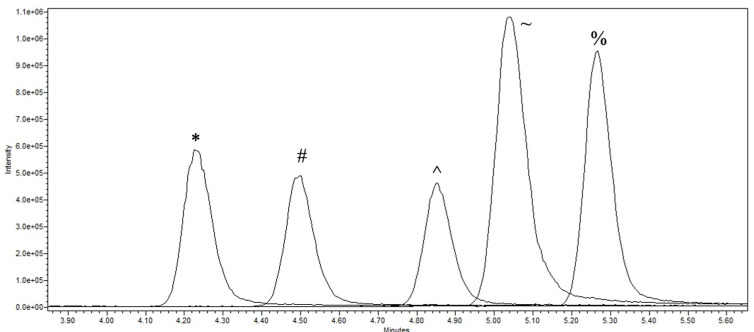
UPLC-MS analysis of BMAA isomers and enantiomers at *m*/*z* 617. The enantiomers/isomers shown are l-2,4-diaminobutyric acid (*), d-2,4-diaminobutyric acid (#), *N*-(2-aminoethyl)glycine (^), β-methylamino-l-alanine (~) and β-methylamino-d-alanine (%).

**Figure 5:**
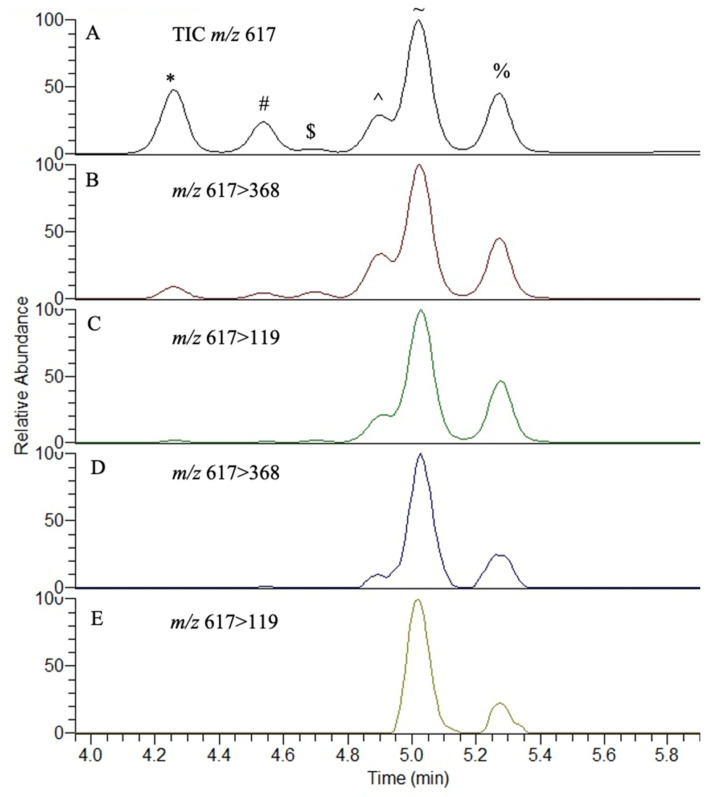
(*S*)-NIFE derivatization and analysis of BMAA enantiomer and isomers, as standards and in an extract of *Lyngbya* spp., showing parent and daughter ions as analyzed by LC-MS/MS. The enantiomers and isomers analyzed were l-2,4-diaminobutyric acid (*), d-2,4-diaminobutyric acid (#), l-*β*-amino-*N*-methylalanine ($), *N*-(2-aminoethyl)glycine (^), *β*-methylamino-l-alanine (~) and *β*-methylamino-d-alanine (%). Panels (**A**–**C**) represent standards and (**D**,**E**) are an extract of cyanobacteria.

BMAA isomers and enantiomers, including BAMA, could be successfully separated after derivatization with (*S*)-NIFE on a C18 column, analyzing double-derivatized enantiomers using single quadrupole mass spectrometry (*m*/*z* 617; Figure 4), and additionally with CID in triple quadrupole mass spectrometry, the single derivatized (*m*/*z* 368) and the underivatized enantiomer/isomer (*m*/*z* 119; Figure 5A–C).

**Figure 6:**
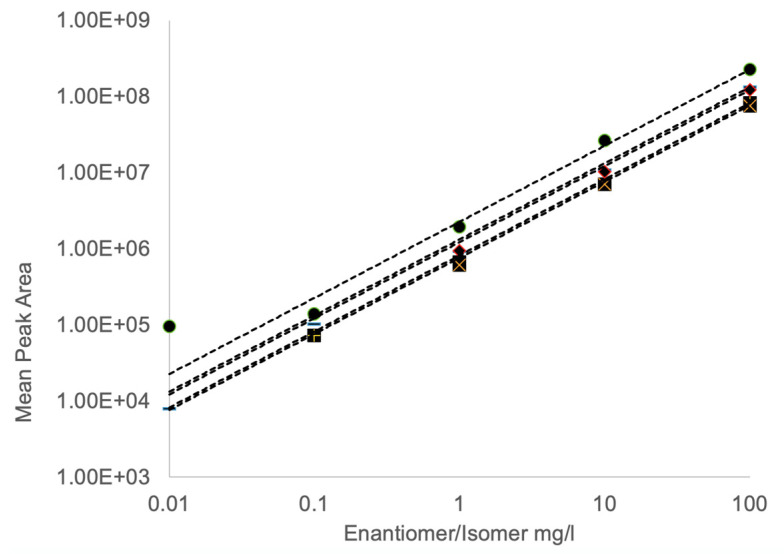
Example standard curves for L-BMAA (•), D-BMAA (−), AEG (+), L-DAB (♦) and D-DAB (×). Samples (0.01 to 100 μg/mL) were derivatized with (*S*)-NIFE and 5 μL injections were assessed by single quadrupole mass spectrometry (n = 6). Regression equations are shown for L-BMAA (y = 2.27 × 10^6^x, R^2^ = 0.999), D-BMAA (y = 1.33 × 10^6^x, R^2^ = 0.999), AEG (y = 8.2 × 10^5^x, R^2^ = 0.999), L-DAB (y = 1.23 × 10^6^x, R^2^ = 0.999) and D-DAB (y = 7.6 × 10^5^x, R^2^ = 0.999).

Using synthetic standards, the linearity of the assay and precision were adequate, with reasonable linearity, intra-day and inter-day precision (Figure 6; Table 1, Table 2 and Table 3). An analysis of the free amino acid extract from an environmental collection of *Lyngbya* after solid-phase extraction showed the presence of L-BMAA and D-BMAA as free amino acids in marine cyanobacterial material, in addition to the likely presence of AEG at free concentrations of 2.03, 1.17 and 0.36 ng/g, respectively. An assessment of SPE methods with separate L- and D-BMAA standards did not show any appreciable racemization to the opposite enantiomer, and differences in recovery were observed with the cation exchange cartridges with recoveries of 82% and 25% for L-BMAA and D-BMAA, respectively. Therefore, the concentrations of free L- and D-BMAA in this *Lyngbya* sample may be an underestimation. Only L-BMAA (0.01 and 0.05 μg/g) and L-DAB (1.0 and 1.4 μg/g) were found as free amino acids in the TCA extractions of CRM-FDMT-1 and CRM-Asp-Mus-d mussel tissues, respectively.

## 3. Discussion

A wide variety of analytical methods have been applied to the detection of BMAA in cyanobacterial and clinical materials [85]. However, such methods are generally unable to distinguish enantiomers (AEG is achiral), and care needs to be taken to ensure that the isomers are also sufficiently separated from each other. Although only the L-enantiomer of BMAA has previously been demonstrated in cyanobacteria [87], the possibility exists that D-BMAA may naturally occur, especially in light of the fact that cyanobacteria are capable of producing a wide range of D-amino acids, such as D-glutamic acid and D-alanine, which are present in the cyclic peptide hepatotoxins, the microcystins [1]. As D-BMAA has also been reported in cycads, of which cyanobacteria are common symbionts [88,110,111], supports the detection of L- and D-BMAA in this study as free amino acids in cyanobacterial extracts (Figure 5). In addition to cyanobacteria and cycads, diatoms, dinoflagellates and chemoheterotrophic bacteria have shown the potential to contain BMAA [101,102,103,104,106,112], and further research should consider the enantiomeric ratios of these isomers within these organisms. As evidence suggests that both L- and D-BMAA can be produced by cyanobacteria, then further research is required to understand how these compounds are produced by cyanobacteria and whether the different enantiomers are interconvertible through, e.g., racemases. At present, the production of (presumably) L-BMAA is considered to be related to the nitrogen status of the cyanobacterial cell, with a temporary increase in its concentration in the cell under conditions of nitrogen starvation, and a subsequent loss after the resumption of nitrogen supply back to the cell [113].

A further possibility for racemization concerns the pH of the environment during cyanobacterial blooms. Although the material used in this study was collected from a marine environment, freshwater cyanobacterial blooms frequently experience high pH due to the removal of carbonic acid during carbon fixation and photosynthesis [114]. If extremes of pH were observed in the cyanobacterial bloom, then potentially, the racemization of certain compounds may occur, as pH and temperature are known to affect amino acid enantiomers and their racemization [92]. In this study, only free amino acids were assessed for enantiomers, as the acid hydrolysis of cyanobacterial material has been shown to racemize BMAA [88]. An assessment of the SPE procedures with L- and D-BMAA standards indicated that little to no racemization occurs. Therefore, the finding of D-BMAA in *Lyngbya* material suggests that this is a naturally occurring enantiomer. Furthermore, as DAB was observed in the extract after AQC derivatization and was not detected after SPE and (*S*)-NIFE derivatization, this may be due to the SPE procedure showing differing abilities to retain AEG and DAB. Further research is required to examine the various SPE phases that are required to obtain other, environmentally relevant neurotoxic amino acids such as DAB.

Previous studies have shown that the chiral separation of BMAA enantiomers is possible and applicable to purified compounds and extracts of organisms such as cycads [86,88]. The only previous study dedicated to examining the presence of L-BMAA in cyanobacteria combined chiral fractionation and measurements of BMAA in the fractions using AQC derivatization and mass spectrometry [87]. Although this time-consuming and labor-intensive procedure showed the presence of L-BMAA as a free amino acid in *Nostoc*, this study aimed to simplify and develop a more user-friendly procedure for routing chiral analysis. Using AQC derivatization, BMAA was shown to be present in the *Lyngbya* extracts, and developing a new analytical method with *S*-NIFE revealed the presence of L- and D-BMAA in the extracts. An analysis of the mussel reference materials only showed the presence of L-BMAA. Although adequate separation of the enantiomers and isomers was achieved, further research could encompass making sure complete separation is achieved and, depending on the matrix, further optimization of the chromatographic system may be necessary to ensure this separation.

Although acute intoxications from naturally occurring BMAA enantiomers and isomers are unlikely, chronic exposure to BMAA has been implicated in diseases such as amyotrophic lateral sclerosis and Alzheimer’s, with chronic dietary exposure to BMAA having been shown to cause neuropathologies that are consistent with these neurodegenerative diseases [115,116]. Ultimately, in order to be able to accurately detect BMAA, methods need to be robust enough to allow for accurate and specific detection. Furthermore, complementary methods can also aid in this endeavor. The analysis of BMAA enantiomers and isomers here have employed two different mass spectrometric methods, using chemical derivatization with AQC and (*S*)-NIFE for isomers and enantiomers, respectively. Using our triple quadrupole analytical method, transitions from parent ions to, e.g., *m*/*z* 171 were used to monitor the different enantiomers at specific retention times. Although this is outside the scope of the current study, future investigations could monitor for the presence of unique daughter ions that may aid in the identification of *S*-NIFE derivatized enantiomers.

The methods developed here have shown that marine samples of cyanobacteria and mollusks contain a number of enantiomers and isomers of BMAA. Research is warranted to better understand the ecological and toxicological effects of these compounds.

## 4. Materials and Methods

### 4.1. Source of Biological and Chemical Materials

The cyanobacterial mat material was collected from the surface of mangrove roots at Matheson Hammock Park, Biscayne Bay, Florida. The material was transported to a laboratory, where it was assessed and confirmed as *Lyngbya* sp. using light microscopy. The material was subsequently frozen, lyophilized and stored at −20 °C, prior to extraction.

Two mussel reference material samples were donated by the National Research Council Canada (reference material NRC-CRM-FDMT-1, NRC-CRM-Asp-Mus-d, Ottawa, ON, Canada) and have previously been shown to contain BMAA (e.g., [99,104]). Consequently, they were included to test for the chirality of the BMAA present within. The amino acid L-BAMA was a gift from Prof. Susan Murch (University of British Columbia, Kelowna, BC, Canada), and the D-BMAA and D-DAB were provided by the investigators (P.B. Wyatt and P.B. Nunn, respectively) at, according to UPLC-UV and/or NMR, >97% purity.

### 4.2. Extraction and Purification of Neurotoxic Amino Acids

The freeze-dried *Lyngbya* material was removed from the freezer and allowed to warm to room temperature. A subsample of the material was removed (24 mg) and placed into a screw-capped glass vial. To this material, 200 µL of 6 M HCl was added and the material was hydrolyzed for 16 h at 110 °C. After cooling, an aliquot (100 µL) was removed and centrifuge-filtered. An aliquot (50 µL) of that filtrate was dried in a Speed Vac (Thermo Scientific Savant, Asheville, NC, USA) on low for 2 h. After drying, the residue underwent solid-phase extraction using C18 and strong cation-exchange cartridges [114]. After elution, the SCX solution was dried in the Speed Vac and resuspended with 1 mL (2 × 500 µL) of 20 mM HCl, which was again dried in the Speed Vac. The residue was resuspended with 100 µL of 20 mM HCl, and a 1/5 dilution was prepared and derivatized with 6-aminoquinolyl-*N*-hydroxysuccinimidyl carbamate (AQC) for analysis by UPLC-MS/MS to verify the presence of total BMAA and isomers [117].

A further subsample (0.7 g) of the cyanobacterial mat material was removed and processed for free amino acids to determine the chirality of the endogenous BMAA. The material was resuspended with 10 mL of 10% TCA (*w*/*v*) in water and sonicated for 20 s. The suspension was allowed to sit at room temperature for 1 h before being placed at 4 °C overnight. The suspension was removed and centrifuged, and the supernatant was aspirated. A second aliquot of 10 mL of 10% (*w*/*v*) TCA was added to the pelleted material and re-sonicated. The suspension was left for 1 h at room temperature before the centrifugation and aspiration of the supernatant. The supernatants were pooled and underwent solid-phase extraction. A total of 9 mL of the pooled supernatant was recovered from the *Lyngbya* material, and this was passed through a 1 g Isolute C18 cartridge, after conditioning with 10 mL of methanol, followed by 10 mL of DQ water. The filtrate from the C18 cartridge was collected, and the cartridge was washed with a further 10 mL of DQ water, which was pooled with the filtrate and freeze-dried. The dried residue was resuspended with 10 mL of 0.1 M HCl for purification with a strong cation-exchange solid-phase extraction cartridge (Strata-X-C, 33 µ 500 mg/3 mL Phenomenex, Torrance, CA, USA). The X-C cartridge was conditioned with 10 mL of methanol, followed by 10 mL of DQ water, and finally 10 mL of 0.1 M HCl. The 10 mL extract was then passed through the cartridge before being washed with 3 mL of 0.1 M HCl and then eluted with 15 mL of 10% NH_4_OH in methanol. The eluate was collected in a rotary evaporation flask and dried in the rotary evaporator. Once dry, the residue was resuspended with 2 × 500 µL of 0.1 M HCl, transferred to an Eppendorf vial and dried in a Speed Vac. Once dry, the residue was resuspended with 500 µL of 20 mM HCl and centrifuged to remove precipitates. The supernatant was removed and stored at –20 °C until it was derivatized with (*S*)-NIFE for chiral analysis.

The freeze-dried mussel samples were also extracted with 10% (*w*/*v*) TCA using sonication in water (3 watts, 60 s) followed by centrifugation (3 min at 5000× *g*), after which the supernatant was removed. This was followed by a second sonication in 10% (*w*/*v*) TCA, and the material was allowed to precipitate at room temperature for one hour. The material was centrifuged (5 min at 5000× *g*) and the supernatants were combined for a total mass to volume ratio of 100 mg/mL. The supernatant was then centrifuge-filtered through a 0.22 μm filter (Ultrafree-MC GV, Sigma-Aldrich, St. Louis, MO, USA) before (*S*)-NIFE derivatization and chiral analysis. 

### 4.3. Analysis of AQC-Derivatized BMAA via UPLC-MS/MS

AQC-derivatized samples of the acid-hydrolyzed *Lyngbya* material were run on a triple quadrupole UPLC-MS/MS system (Thermo Scientific Finnigan TSQ Quantum UltraAM, San Jose, CA, USA) after separation, using a Waters Acquity-UPLC system comprising a Binary Solvent Manager, a Sample Manager and a Waters AccQTag Ultra column (part# 186003837, 2.1 × 100 mm, 1.7 μm), maintained at 55 °C. Separation was achieved with gradient elution at a flow of 0.65 mL/min, with solvents of 0.1% (*v*/*v*) formic acid in water (eluent A) and 0.1% (*v*/*v*) formic acid in acetonitrile (eluent B): gradient of 99.1%A @ 0 min; 99.1%A curve 6 @ 0.5 min; 95%A curve 6 @ 2 min; 95%A curve 6 @ 3 min; 90%A curve 8 @ 5.5 min; 15%A curve 6 @ 6 min; 15%A curve 6 @ 6.5 min; 99.1%A curve 6 @ 6.6 min; 99.1%A curve 6 @ 8 min. To the HESI (heated electrospray ionization) probe, nitrogen gas was supplied with a nebulizing pressure of 40 psi and a vaporizer temperature of 400 °C. The mass spectrometer was then operated under the following conditions: the capillary temperature was set to 270 °C, the capillary offset was set to 35, the tube lens offset was set to 110 and the aux gas pressure was set at 35, with a spray voltage of 3500 V, a source collision energy of 0, and a multiplier voltage set to −1719 V. During the equilibration and cleaning phases of the gradient, a divert valve was used. Using 100% argon, the second quadrupole was pressurized to 1.0 mtorr. During the BMAA analysis of the cyanobacterial and mussel extracts, collision-induced dissociation (CID) was achieved in the second quadrupole using the following transitions: *m*/*z* 459 to *m*/*z* 119 CE 21; *m*/*z* 459 to *m*/*z* 289 CE 17; and *m*/*z* 459 to *m*/*z* 171 CE 38. The resultant product ions were scanned using the third quadrupole, detected, and their relative abundances were quantified in comparison with the AQC-derivatized *N*-(2-aminoethyl)glycine (AEG), L-BMAA and l-2,4-diaminobutyric acid (DAB) standards. For the cyanobacterial extracts, the precursor ion of *m*/*z* 459 was monitored for CID daughter ions of *m*/*z* 171, 289, 119, 258 (BMAA), 214 (AEG) and 188 (DAB), with the last three daughter ions being qualifier ions for the different isomers.

### 4.4. Analysis and Partial Validation of (S)-NIFE Derivatized Standards and Cyanobacterial Samples

The SPE cleaned and purified extract of *Lyngbya* was derivatized with (*S*)-NIFE (*N*-(4-nitrophenoxycarbonyl)-l-phenylalanine 2-methoxyethyl ester) in a 150 µL PCR tube, with a 12.5 µL sample added to 12.5 µL of 0.2 M sodium tetraborate and mixed, before the addition of 12.5 µL of (*S*)-NIFE (Santa Cruz Biotechnology (sc-253524), Dallas, TX, USA, 5 mg/mL in acetonitrile). The filtered extracts of the mussel tissues were derivatized with (*S*)-NIFE using a 10 µL sample added to 1.5 µL of 19.3 M NaOH plus 28.2 µL of 0.2 M sodium tetraborate and mixed, before the addition of 6.25 µL of *S*-NIFE. After mixing, the samples were left at room temperature for 20 min, before the addition of 4 M HCl (2.5 µL for *Lyngbya* and 5.0 µL for mussel tissues) and finally, DQ water (50 µL for *Lyngbya* and 75 µL for mussel tissues). After derivatization, the samples were transferred to a total recovery UPLC vial and placed in the sample manager, maintained at 4 °C. From these vials, 5 µL injections were performed using partial loop injection and assessed by single (Waters EMD1000, Milford, MA, USA) or triple quadrupole mass spectrometry (Thermo TSQ Quantiva or Thermo TSQ Quantum UltraAM, Waltham, MA, USA). Using the single quadrupole mass spectrometer, a preliminary validation of the *S*-NIFE method was carried out, examining the retention time range, linearity, the limits of detection and quantification and the inter- and intra-day precision for the enantiomers/isomers, and all calculations were performed using Microsoft Excel. The sample was supplied to the mass spectrometers using a Waters Acquity ultra-high-pressure liquid chromatographic system, with a binary solvent manager and sample manager, as well as a Phenomenex Kinetix 1.7 µ 100 Å C18 column (100 × 2.1 mm for *Lyngbya)* and a Thermo Hypersil Gold (Waltham, MA, USA) 1.9 µ 100 Å C18 column (100 × 2.1 mm for mussel tissue), maintained at 55 °C. The separation of the available BMAA isomers and enantiomers (L-BMAA, D-BMAA, L-BAMA, L-DAB, D-DAB, AEG) was performed after derivatization with (*S*)-NIFE, with the exception that after derivatization, the standards received 97.5 µL of DQ water (*versus* 50 or 75 µL of DQ water for the samples). The flow was maintained at 0.2 mL/min, with a gradient of 37–58% B (acetonitrile + 0.1% (*v*/*v*) formic acid) and curve 10 over 5 min (curve 10 for *Lyngbya* and curve 9 for mussel tissues), before a wash of 100% B from 5.1 to 5.5 min, before returning to the starting conditions of 63% A (water + 0.1% (*v*/*v*) formic acid) and 37% B at 5.6 min, with a total run time of 8 min. The double-derivatized BMAA enantiomers were monitored at *m*/*z* 617.4, with transitions from *m*/*z* 617 > 368 and 617 > 119 (NCE = 19) for the single-derivatized and underivatized isomers, respectively.

## Figures and Tables

**Table 1:** Coefficients of variation regarding retention time and area for L-BMAA, its isomers and enantiomers (n = 6).

		L-BMAA		D-BMAA		AEG		L-DAB		D-DAB	
Conc (μg/mL)	ng on Column	RT %CV	Area %CV	RT %CV	Area %CV	RT %CV	Area %CV	RT %CV	Area %CV	RT %CV	Area %CV
0.1	0.5	0.18	12.32	0.16	17.03	0.27	18.73	ND	ND	ND	ND
1	5	0.40	22.93	0.20	12.02	0.25	10.09	0.24	11.65	0.25	9.42
10	50	0.26	25.70	0.25	29.06	0.24	14.14	0.26	12.68	0.23	10.66
100	500	0.21	13.32	0.22	8.58	0.23	7.80	0.23	7.96	0.26	5.73

ND, not detectable.

**Table 2:** Variations in retention time for BMAA isomers and enantiomers as analyzed by UPLC-MS, along with limits of detection and quantification (n = 6).

	L-BMAA	D-BMAA	AEG	L-DAB	D-DAB
Conc (μg/mL)	RT range (min)	RT range (min)	RT range (min)	RT range (min)	RT range (min)
0.1	5.098–5.127	5.268–5.292	4.822–4.856	4.211–4.240	4.482–4.499
1	5.096–5.147	5.269–5.298	4.824–4.853	4.223–4.237	4.479–4.507
10	5.062–5.092	5.265–5.298	4.827–4.856	4.213–4.242	4.478–4.501
100	5.028–5.058	5.255–5.268	4.826–4.851	4.211–4.239	4.472–4.499
LOD (μg/mL)	0.0013	0.0025	0.0031	0.0025	0.0033
LOQ (μg/mL)	0.0042	0.0085	0.0103	0.00846	0.0111

**Table 3:** Examples of linearity, intra-day and inter-day precision for BMAA enantiomers and isomers.

Enantiomer	Linearity (μg/mL)	Intra-Day Precision (%)	Inter-Day Precision (%)
L-BMAA	0.1–100	87–112	85–117
D-BMAA	0.1–100	95–104	88–117
AEG	0.1–100	95–104	91–112
L-DAB	0.1–100	90–109	86–112
D-DAB	0.1–100	88–111	90–110

## Data Availability

Data may be made available after contact with the authors.
